# Compound Meta-Optics for Advanced Optical Engineering

**DOI:** 10.3390/s26030792

**Published:** 2026-01-24

**Authors:** Hak-Ryeol Lee, Dohyeon Kim, Sun-Je Kim

**Affiliations:** 1 School of Electrical Engineering, Soongsil University, 369 Sangdoro, Dongjak-Gu, Seoul 06978, Republic of Korea; ktxgumdo@naver.com (H.-R.L.); dohyeon9816@naver.com (D.K.); 2 Future Technology Research Institute, Soongsil University, 369 Sangdoro, Dongjak-Gu, Seoul 06978, Republic of Korea

**Keywords:** metasurface, metalens, meta-optics, compound meta-optics, aberration, information capacity, imaging, display, sensing, meta-hologram, optical computing

## Abstract

Compound meta-optics, characterized by the unprecedented complex optical architectures containing single or multiple meta-optics elements, has emerged as a powerful paradigm for overcoming the physical limitations of single-layer metasurfaces. This review systematically examines the recent progress in this burgeoning field, primarily focusing on the development of high-performance optical systems for imaging, display, sensing, and computing. We first focus on the design of compound metalens architectures that integrate metalenses with additional elements such as iris, refractive optics, or other meta-optics elements. These configurations effectively succeed in providing multiple high-quality image quality metrics simultaneously by correcting monochromatic and chromatic aberrations, expanding the field of view, enhancing overall efficiency, and so on. Thus, the compound approach enables practical applications in next-generation cameras and sensors. Furthermore, we explore the advancement of cascaded metasurfaces in the realm of wave-optics, specifically for advanced meta-holography and optical computing. These multi-layered systems facilitate complex wavefront engineering, leading to significant increases in information capacity and functionality for security and analog optical computing applications. By providing a comprehensive overview of fundamental principles, design strategies, and emerging applications, this review aims to offer a clear perspective on the pivotal role of compound meta-optics in devising and optimizing compact, multifunctional optical systems to optics engineers with a variety of professional knowledge backgrounds and techniques.

## 1. Introduction

The relentless pursuit of miniaturization in modern information technology has necessitated a fundamental transformation in optical engineering. From ubiquitous smartphone cameras to emerging augmented and virtual reality (AR/VR) interfaces and autonomous sensing systems, there is a critical demand for optical components that are not only compact and lightweight but also capable of delivering high-performance imaging [1,2,3,4,5]. Traditional refractive optics, which rely on the accumulation of phase via propagation through bulky glass or plastic media, face inherent physical limitations in size, weight, and optical functions. To overcome these constraints, metasurfaces, ultra-thin arrays of subwavelength nanostructures, have emerged as a paradigm-shifting solution, offering the ability to manipulate the amplitude, phase, polarization, and spectrum of light with unprecedented flexibility at the subwavelength scale [4,5,6].

In the initial stages of development, research primarily focused on single-layer metasurfaces. These devices demonstrated remarkable capabilities, including flat lensing, beam steering, and holographic projection. However, despite their promise, single-layer metalenses are frequently plagued by fundamental optical limitations, most notably strong chromatic aberrations, limited field of view (FOV), and monochromatic Seidel aberrations (spherical aberration, coma, astigmatism, field curvature, and distortion) when scaling to elements with high numerical aperture (NA), broad bandwidth, or large lens aperture [7,8,9,10,11,12,13,14,15,16,17,18,19,20]. While various dispersion-engineering techniques have been proposed to mitigate these issues, the physical degrees of freedom available in a single planar element are often insufficient to simultaneously correct multiple aberrations across a broad spectral range and wide angular field.

To overcome these limitations, the field is currently witnessing a rapid evolution toward compound meta-optics [21,22,23]. Analogous to the historical development of refractive optics—where compound objectives replaced singlet lenses to achieve high-quality imaging—compound meta-optics involves the strategic cascading of multiple metasurfaces or the integration of metasurfaces with refractive elements. This architecture introduces additional degrees of freedom, allowing for precise aberration correction and system-level optimization that is unattainable with a single surface.

This review systematically examines the recent progress in compound meta-optics, with a predominant focus on its transformative role in advanced imaging [24,25,26], display [27,28,29,30], and sensing and computing systems [31,32,33,34]. Unlike prior reviews that may broadly categorize metasurface applications, we specifically address the engineering challenges and solutions required to transition metalenses from laboratory curiosities to viable, high-performance optical systems.

We first establish the fundamental principles governing meta-optics and cascaded meta-optics systems, exploring how interlayer propagation and alignment affect optical performance. Subsequently, we delve into the core design strategies employed to correct chromatic and monochromatic aberrations, enabling full-color, diffraction-limited imaging. This review then highlights State-of-the-Art applications across three key domains:

1. Imaging systems: Including miniaturized cameras for mobile devices and endoscopic bio-imaging.
2. Display technologies: Focusing on near-eye or 3-dimensional display systems that require high-level image quality and compact form factors simultaneously.
3. Sensing and computing interfaces: Covering advances in wavefront sensing and various optical computing technologies.

By providing a comprehensive overview of the design methodologies, this article aims to offer a roadmap for the future of compact, high-performance optical system design. The other parts of this review are organized as follows. We begin by establishing the fundamental principles of compound meta-optics. This review then progresses to specific implementations, specifically detailing cascaded metalens systems for high-performance imaging, and cascaded metasurface architectures for advanced meta-holography and analog optical computing in optical neural networks. Then, this paper concludes with a summary of current achievements and future directions.

## 2. Background Knowledges of Compound Meta-Optics

### 2.1. Principles of Phase-Gradient Metasurfaces

**Figure 1:**
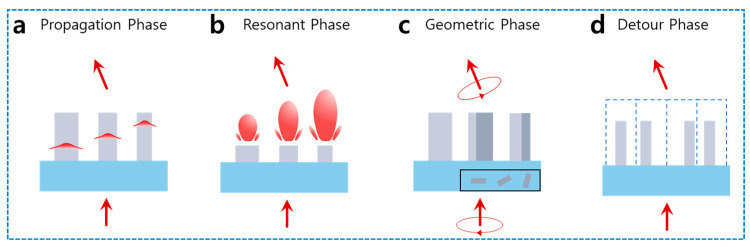
Phase modulation methods of meta-atoms [36]. (**a**) Propagation phase based on spatially varying cross-section of waveguiding meta-atoms. (**b**) Resonant phase originating from multipolar Mie scattering, depending on the geometric parameters of meta-atoms and wavelength of incident light. (**c**) GP method based on rotation of meta-atoms and circular polarization. (**d**) Detour phase using the 1st-order diffraction and transversal shift of meta-atom from the center of a unit cell.

The mechanisms for imposing abrupt phase discontinuities across a subwavelength interface can be broadly categorized based on how the nanostructures interact with the incident light field (Figure 1). The first category relies on tuning the dimensions of the meta-atoms. The propagation phase operates on the waveguide principle, where the phase accumulation is determined by the effective refractive index of the nanopillars (Figure 1a) [18,35,36]. By varying the cross-sectional geometry or diameter of high-aspect-ratio structures, light is confined within the waveguides, allowing for a smooth phase evolution proportional to the structure’s height. In contrast, the resonant phase exploits the strong interaction between light and scatterers at specific frequencies, such as Mie or Fabry–Perot resonances. This approach induces rapid phase shifts near the resonance peak, enabling full 2π phase coverage with relatively thin structures, although it is often accompanied by narrower operating bandwidths due to the dispersive nature of the resonance (Figure 1b).

Alternatively, phase modulation can be achieved through the spatial orientation or arrangement of identical meta-atoms, often decoupling the phase profile from the spectral response. The geometric phase (GP), also known as the Pancharatnam–Berry phase, arises from the in-plane rotation of anisotropic nanofins (Figure 1c) [17]. This mechanism imparts a phase shift equal to twice the rotation angle for circularly polarized light, offering a robust and dispersionless phase response that is ideal for broadband applications. Meanwhile, the detour phase draws inspiration from classical blazed gratings (Figure 1d) [37]. It generates phase gradients through the precise lateral displacement of scattering elements within a unit cell. By shifting the position of the meta-atom relative to the periodicity, an optical path difference is created, allowing for flexible wavefront engineering without relying solely on the local resonance or polarization state of the incident light.

### 2.2. Principles of Camera, Microscope, and Telescope

**Figure 2:**
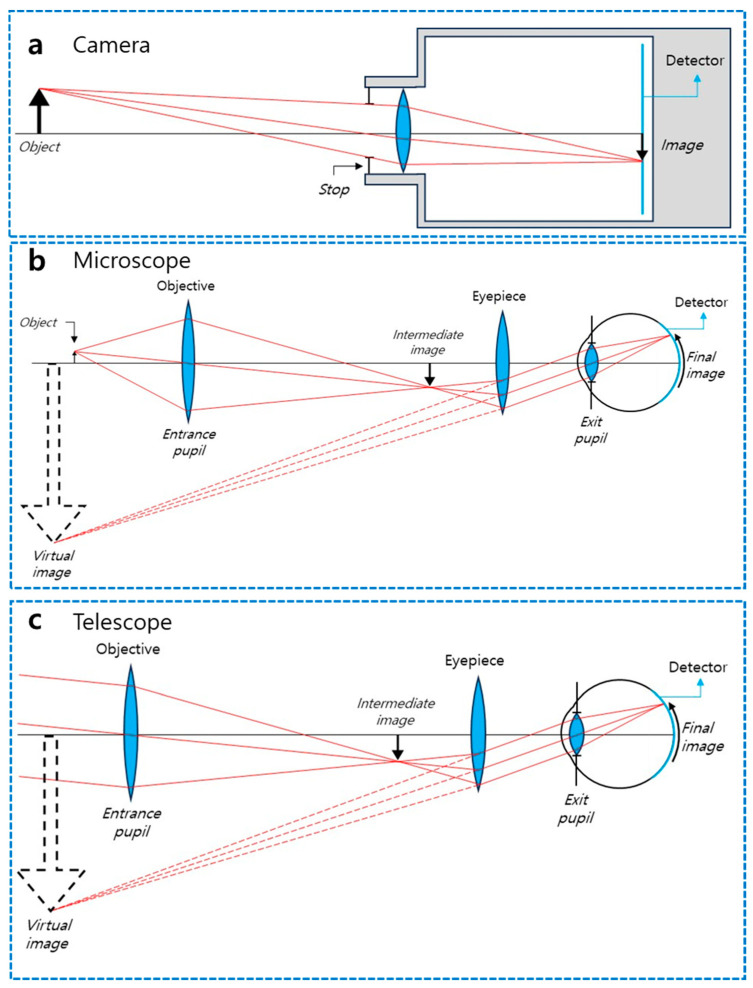
Schematic diagrams illustrating the traditional design rules of camera, microscope, and telescope based on refractive optics [38]. (**a**) Camera system consisting of a front stop, an objective lens, and a detector array. (**b**) Microscope system consisting of an objective lens, an eyepiece, and an eye. (**c**) Telescope system consisting of an objective lens, eyepiece lens, and an eye.

The design of compound meta-optical systems frequently draws inspiration from classical geometric optics, adopting established architectures to achieve specific imaging goals (Figure 2). The most fundamental configuration is the camera system, illustrated in Figure 2a. This setup typically operates on a finite-conjugate principle (or infinite-conjugate for distant scenes), where an aperture stop limits the incoming light and a lens focuses the rays from an object onto a sensor plane to form a real, inverted image. In the context of meta-optics, this architecture serves as the baseline for metalens cameras, where the bulky refractive lens is replaced by a single or multi-layer metasurface to correct monochromatic and chromatic aberrations while drastically reducing the system’s physical track length.

For imaging microscopic features, the compound microscope architecture, shown in Figure 2b, provides the necessary magnification through a cascaded lens system. Light from a nearby object is first collected by an objective lens, which forms a magnified real intermediate image. This intermediate image is then further magnified by an eyepiece to create a virtual image for the observer or a final real image on a detector. In meta-optical implementations, this two-stage magnification process is realized by stacking metasurfaces vertically. This approach allows for high-NA imaging and precise wavefront control in a compact form factor, effectively replacing the complex and heavy objective lens assemblies found in traditional microscopes.

Conversely, the telescope system outlined in Figure 2c is designed to resolve distant objects. Unlike the microscope, the telescope collects collimated rays from infinity. The objective lens focuses these parallel rays to form an intermediate image, which is then viewed or re-collimated by the eyepiece to provide angular magnification. This configuration is particularly significant for meta-optical beam expanders and LIDAR systems. By utilizing the principles of the Keplerian or Galilean telescope designs, cascaded metasurfaces can steer, expand, or focus beams from distant sources with high efficiency, all within a planar architecture that eliminates the bulk associated with conventional telescopic tubes.

### 2.3. Brief History of Compound Meta-Optics

**Figure 3:**
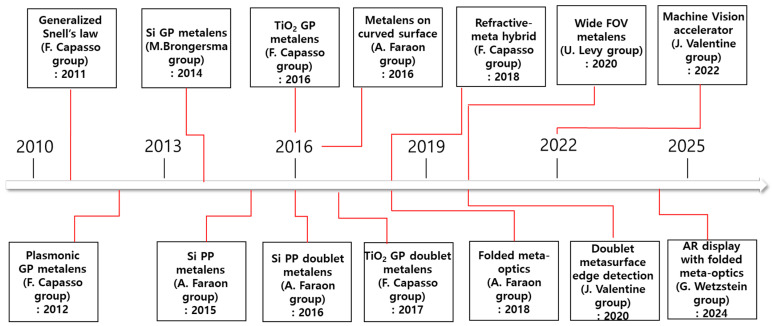
Timeline of meta-optics and compound meta-optics with the milestone papers [6,17,18,39,40,41,42,43,44,45,46,47,48,49].

The chronological evolution of meta-optics, as depicted in the timeline described in Figure 3, originates from the seminal formulation of the Generalized Snell’s Law by the Capasso group (N. Yu et al. (2011)) [6]. This fundamental breakthrough established the theoretical capability to arbitrarily mold optical wavefronts using subwavelength phase discontinuities, triggering a surge of pioneering research into single-layer metasurfaces. Early experimental milestones from leading research groups—including the groups led by F. Capasso, M. Brongersma, and A. Faraon—demonstrated the feasibility of planar optics, successfully implementing flat lenses that operate in regimes previously dominated by bulk refractive components, via theory, numerical analysis, and experiment [17,18,39]. These foundational studies of two-dimensional optical metamaterial were pivotal in verifying the efficacy of abrupt phase manipulation at the interface, thereby setting the stage for the transition from simple wavefront shaping to functional optical elements.

As the field matured, the focus shifted toward overcoming the intrinsic limitations of single-layer devices, such as severe monochromatic aberrations and restricted FOV, leading to the era of compound meta-optics. The timeline highlights this paradigm shift through key developments, including the realization of aberration-correcting doublet metalenses and hybrid architectures that synergize refractive optics with metasurfaces to enhance optical performance. Furthermore, the integration of system-level components, such as aperture stops for wide-FOV imaging (iris-integrated metalenses), marked a significant leap in engineering complexity. These advancements have collectively propelled the technology toward practical utility, enabling the diverse array of compound metalens applications currently emerging in advanced imaging, sensing, and augmented reality systems. In the next section, recent advances of cascaded metalens systems which adopted the abovementioned three fundamental imaging systems are introduced and discussed.

## 3. Cascaded Metalens System with Advanced Performance

### 3.1. Metalens Combined with an Iris

As the first category of the cascaded metalens system, one can look into the metalens camera architecture demonstrated by combination of an additional iris and a metalens. It is a widely adopted strategy that involves placing an aperture stop (iris) in front of the metalens, analogous to the classical “landscape lens” architecture to address the severe off-axis aberrations inherent in single-element optics. This configuration restricts the ray bundle for a given angle of incidence to a specific sub-region of the lens, thereby effectively manipulating the system’s symmetry. In this setup, the metalens is typically designed with a quadratic phase profile rather than the standard hyperboloidal one. The quadratic phase distribution possesses a unique property of symmetry transformation, where the rotational symmetry of oblique illumination is converted into the translational symmetry of the focal spot. By carefully optimizing the distance between the stop and the metalens, the coma and astigmatism can be significantly suppressed, allowing the system to achieve a wide FOV with diffraction-limited performance [47,50,51,52,53,54,55,56,57,58,59,60,61].

**Figure 4:**
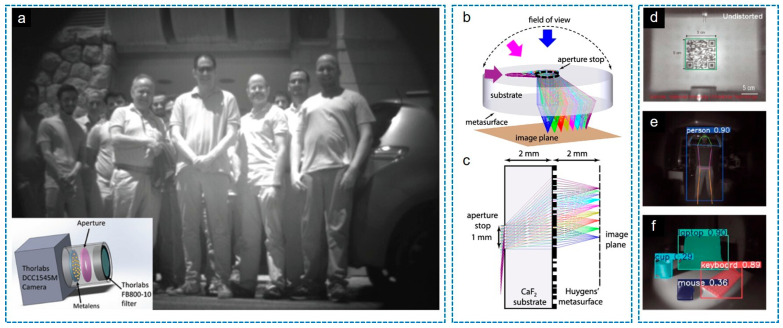
Recent advances of wide FoV imaging applications using an iris combined with a single metalens. (**a**) Near-IR camera based on a Huygens metalens and an iris [47]. (**b**) 3D and (**c**) 2D ray tracing results and schematic diagrams of IR Fisheye metalens [50]. (**d**–**f**) Machine vision tasks [52]. Figure adapted with permission from (**a**) Ref. [47], Copyright 2020, U. Levy et al., licensed under CC BY 4.0.; (**b**,**c**) Ref. [50], Copyright 2020, American Chemical Society; and (**d**–**f**) Ref. [52], Copyright 2025, S. Li et al., licensed under CC BY 4.0.

In particular, several representative studies have successfully demonstrated this aperture-coupled architecture. As described in Figure 4a, Engelberg et al. [47] utilized an air gap between the aperture stop and a quadratic Huygens metalens, achieving a near-diffraction-limited FOV of approximately 40° for outdoor near-infrared imaging. To further expand the FOV to the ultra-wide regime, recent works have replaced the air gap with a high-index dielectric substrate, which helps match the ideal focal offset condition (*s*(*θ*) ≈ *f*sin *θ*). Shalaginov et al. [50] reported a single-element fisheye metalens integrated on a thick sapphire substrate, realizing a panoramic FOV close to 180° (Figure 4b,c). Similarly, Zhang et al. [51] demonstrated an extreme-angle imaging system on a silica substrate using optimized streamline structures, achieving a record FOV of 178° with high efficiency and uniformity. These works collectively highlight the efficacy of the landscape-lens-inspired meta-optics in realizing compact, wide-angle imaging systems.

A compelling demonstration of metalens-based machine vision was recently presented by Li et al., who integrated a high-resolution, wide-FOV (100°) metalens with the advanced YOLO11 object detection framework [52]. By employing a silicon-based landscape lens architecture with a front aperture stop, they achieved near-diffraction-limited imaging at a wavelength of 633 nm within an ultra-compact volume of just 0.04 cm^3^. As shown above in Figure 4d–f, the study experimentally validated the system’s versatility across diverse vision tasks, successfully performing close-range QR code scanning, medium-range object classification, and long-range human-pose estimation. This work confirms that lightweight meta-optics can effectively replace bulky traditional lenses in next-generation intelligent recognition systems without compromising the image quality required for deep-learning algorithms.

Beyond general-purpose computer vision, wide-angle metalenses are finding critical utility in specialized fields. In the medical field, endoscopic imaging [53] utilizes the wide FOV to visualize internal organs with minimal invasiveness. Furthermore, automotive monitoring (e.g., driver-fatigue detection) and AR devices leverage the planar and lightweight nature of metalenses to integrate wide-angle sensing into wearable or embedded systems, where space is strictly limited.

### 3.2. Compound System with Cascaded Metalenses

**Figure 5:**
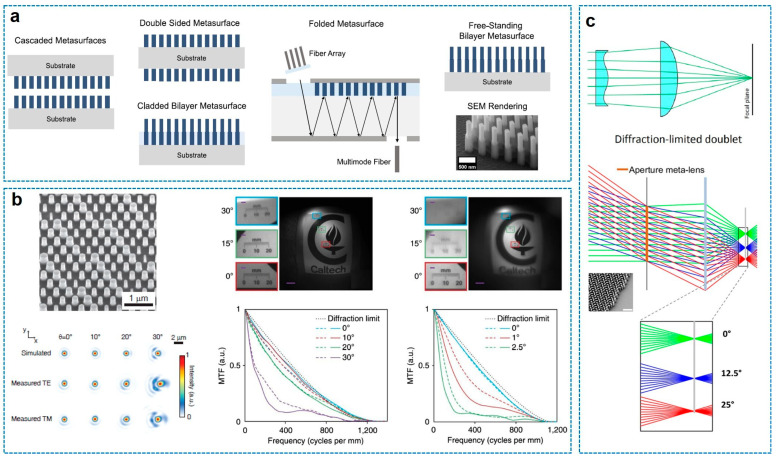
Compound meta-optical systems constructed by cascade of multiple metalenses. (**a**) Category of cascaded systems [22]. (**b**) Doublet a-Si metalens-based coma-corrected miniature camera in the near-infrared using propagation-phase method [41]. (**c**) Doublet visible TiO_2_ metalens using GP method to correct coma aberration. The doublet is optimized as the sequential cascade system of a Schmidt plate-like metalens and a plano-convex-like metalens [43]. Figure adapted with permission from (**a**) Ref. [22], Copyright 2025, A. H. Dorrah; (**b**) Ref. [41], Copyright 2016, A. Arbabi et al., licensed under CC BY 4.0.; and (**c**) Ref. [43], Copyright 2017, American Chemical Society.

To overcome the severe off-axis aberrations inherent in single-layer metasurfaces, recent research has pivoted toward cascaded architectures that introduce additional degrees of freedom for wavefront engineering. As illustrated in Figure 5a, these multi-element systems can be realized through various configurations, including vertically stacked substrates, double-sided patterning on a single substrate, folded optical paths within a waveguide, or free-standing bilayer structures. Among these, the monolithic double-sided metalens doublet for a wide-FOV camera has emerged as a robust platform for correcting multiple monochromatic aberrations, such as coma and astigmatism, over a wide FOV [41,43,62,63,64,65,66,67].

A seminal implementation of this architecture is the miniature planar camera demonstrated by A. Arbabi et al. [41], shown in Figure 5b. Operating in the near-IR region (850 nm), this doublet utilizes high-contrast amorphous silicon nanoposts (propagation-phase method) patterned on both sides of a fused silica substrate. The monochromatic doublet camera functions as a fisheye objective with a low f-number of 0.9 and an FOV larger than 60° by 60°. By dividing the optical power and aberration correction duties between the two surfaces—essentially acting as a correcting plate and a focusing lens—the system achieves near-diffraction-limited focusing across a wide angular range, effectively mitigating the off-axis distortions that plague singlet designs.

Extending this concept to the visible spectrum, Groever et al. [43] demonstrated a metalens doublet using TiO_2_ nanofins based on the GP principle, as depicted in Figure 5c. Drawing inspiration from the classical Chevalier landscape lens, this key idea of the design is to design the first surface (aperture metalens) that functions analogously to a Schmidt plate and the aperture stop of the system, correcting spherical aberrations for oblique incidence, while the second surface is set to be the focusing metalens. This configuration allows for diffraction-limited imaging with an NA of 0.44 and an FOV of 50° at a wavelength of 532 nm. The principle and performance of this work is similar to the seminal work by A. Arbabi et al. [41], but this work provide physical insight in comparison to the well-known lens design of the Chevalier landscape lens. These studies collectively establish that cascading metasurfaces on a monolithic substrate is a scalable and effective strategy for realizing high-performance, wide-angle meta-optics.

**Figure 6:**
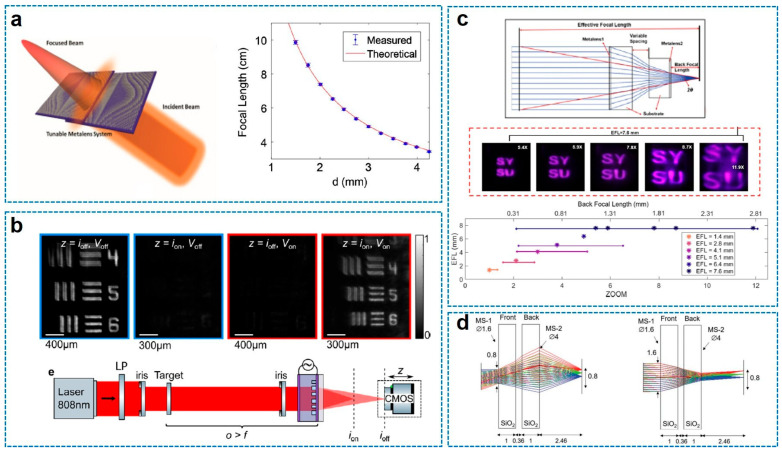
Recent studies on zoom metalens applications. (**a**) Mechanical translation (transversal)-based zoom doublet metalens [68]. (**b**) Electrically tunable LC-coupled metalens [69]. (**c**) Mechanical translation (optic axis)-based zoom doublet metalens [70]. (**d**) Polarization-controlled parfocal zoom lens doublet [71]. Figure adapted with permission from (**a**) Ref. [68], Copyright 2018, S. Colburn; (**b**) Ref. [69], Copyright 2025, American Chemical Society; (**c**) Ref. [70], Copyright 2024, American Chemical Society; and (**d**) Ref. [71], Copyright 2022, F. Yang et al., licensed under CC BY 4.0.

One of the most impactful applications of compound meta-optics is the realization of ultrathin zoom lenses [68,69,70,71,72,73,74,75,76,77,78,79,80]. Unlike conventional bulky zoom objectives that require complex mechanisms, meta-optics systems can achieve varifocal functionality through novel tuning mechanisms. Figure 6 illustrates four representative approaches, ranging from lateral actuation (Figure 6a) and liquid crystal (LC)-enabled tunable birefringence (Figure 6b) to axial displacement (Figure 6c) and polarization multiplexing (Figure 6d).

First, the mechanical lateral actuation method was demonstrated by S. Colburn et al. [68], utilizing the Alvarez lens principle (Figure 6a). Unlike standard lenses that require axial movement, the Alvarez design consists of two cascaded metasurfaces, each imparting a phase profile of cubic polynomial function with opposite sign. When these plates are laterally displaced (in *x*-direction) relative to each other, the cubic terms cancel out, yielding a quadratic phase profile corresponding to a lens, where the focal length is inversely proportional to the lateral displacement. Physically, this method allows for a wide tuning range (200% tunability of focal length) with minimal mechanical motion. In terms of fabrication, this study is particularly notable for utilizing stepper photolithography rather than electron-beam lithography, enabling the mass production of large-area (1 cm^2^) SiNx metasurfaces compatible with CMOS foundries.

The second example is to use LC. To eliminate mechanical motion entirely, M. Bosch et al. [69] proposed an electrically tunable varifocal metalens by integrating a single-layer metasurface with LCs (Figure 6b). The physical principle relies on the birefringence of nematic LCs; by applying an AC voltage across ITO electrodes, the orientation of the LC molecules rotates, continuously modifying the environmental refractive index surrounding the meta-atoms. This index change shifts the resonant modes of the a-Si nanopillars, thereby modulating the imparted phase profile and focal length in real time. The device was fabricated by encapsulating the a-Si metasurface within a 5 um thick LC cell sandwiched between glass substrates, demonstrating that active modulation can be achieved in an ultrathin form factor without any moving parts.

On the other hand, J. Zhang et al. [70] realized a fully meta-optical zoom lens with an impressive 11.9 zoom range using axial actuation (Figure 6c). This system mimics the classical mechanical zoom architecture by changing the axial distance between a convex metalens and a concave metalens. The key physical insight here is the utilization of a polynomial phase profile (mimicking an aspheric lens) rather than a standard hyperbolic profile. This optimization significantly minimizes spherical aberrations and extends the depth of focus (DOF), allowing for continuous zoom over a wide range with only minute axial movements (0.1 mm). The device was constructed using crystalline silicon (c-Si) on sapphire substrates via a transfer process, ensuring high transmission efficiency and precise phase delivery at near-infrared wavelengths.

Finally, F. Yang et al. [71] demonstrated a reconfigurable parfocal zoom metalens doublet based on polarization multiplexing (Figure 6d). This system achieves a discrete 10-times optical zoom by switching between two orthogonal polarization states, corresponding to a “wide-angle” mode and a “telephoto” mode. The physical ingenuity lies in the design of the front metasurface, which functions simultaneously as a tunable lens and a variable aperture to maintain a constant focal plane position (parfocality) despite the change in effective focal length. The doublet was fabricated using electron-beam lithography to pattern amorphous silicon nanoposts on fused silica substrates, with the meta-atoms carefully dimensioned to provide independent phase control for *x*- and *y*-polarized light, effectively embedding two distinct optical systems into a single physical stack.

**Figure 7:**
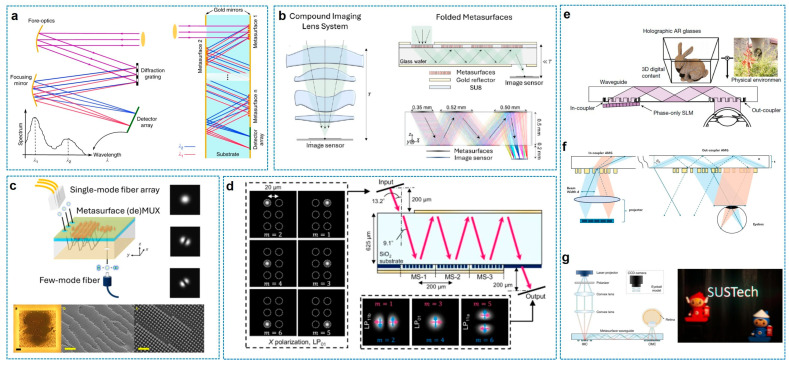
Folded meta-optics-based compact cascaded meta-optics systems. (**a**) Spectrometer based on dispersive camera [45]. (**b**) Compact camera based on 4 different metalenses [81]. (**c**) Fiber-mode demultiplexer [82]. (**d**) Generalized vectorial mode converter based on triplet metalens [83]. (**e**) High-resolution holographic AR glass with waveguide coupled to two metagratings [49]. (**f**,**g**) Wide-FoV AR glass with two inverse-designed metagrating couplers [84,85]. Figure adapted with permission from (**a**) Ref. [45], Copyright 2018, M. S. Faraji-Dana; (**b**) Ref. [81], Copyright 2024, Y. Kim et al., licensed under CC BY 4.0.; (**c**) Ref. [82], Copyright 2022, American Chemical Society; (**d**) Ref. [83], Copyright 2025, G. Soma et al., licensed under CC BY 4.0; (**e**) Ref. [49], Copyright 2024, M. Gopakumar et al., licensed under CC BY 4.0; and (**g**) Ref. [85], Copyright 2025, Z. Tian et al., licensed under CC BY 4.0.

Figure 7 describes the next category, named “folded meta-optics with a waveguide”. In this configuration, instead of propagating through free space, light is guided inside a transparent substrate (like fused silica or high-index glass) via total internal reflection (TIR) or reflective mirror coatings. Metasurfaces are patterned on the substrate surfaces to manipulate the wavefront (phase, amplitude, and polarization) at each interaction point. Folded meta-optics technology is demonstrating exceptional versatility across diverse fields such as sensing, imaging, computing, and communications by enabling extreme miniaturization and device integration [45,49,81,82,83,84,85,86,87].

In the realm of sensing, M. S. Faraji-Dana et al. demonstrated the potential for dramatic miniaturization of conventional tabletop instruments by realizing an ultra-compact spectrometer (Figure 7a). By dispersing and focusing light within a 1 mm thick glass slab, they achieved a spectral resolution of approximately 1.2 nm within a volume of merely 7 mm^3^ [45]. In the imaging domain, Y. Kim et al. proposed a wide-FOV meta-camera system with ultrathin thickness by diagonally folding the optical path inside a glass wafer (Figure 7b). This approach achieved a track length of 0.7 mm—half the effective focal length of 1.4 mm—and delivered quasi-diffraction-limited imaging performance at a wavelength of 852 nm [81].

For optical communications, J. Oh et al. introduced a mode-division multiplexing (MDM) device utilizing a metasurface cavity (Figure 7c). This technology efficiently converts Gaussian beams from single-mode fibers into specific spatial modes (e.g., LP_01_ and LP_11_) without requiring external optics [82]. Furthermore, Soma et al. implemented a complete vectorial mode converter by integrating a multi-layer metasurface architecture into a single chip (Figure 7d). This device simultaneously converts multiple orthogonal input modes into desired output vectorial modes with arbitrary spatial and polarization profiles, validating the potential for universal linear optical transformations on a chip [83].

Folded meta-optics with a glass waveguide are also emerging as a critical technology for AR glass combiners, a key enabler of next-generation spatial computing. Recently, M. Gopakumar et al. achieved high-quality, full-color 3D holographic images with accurate depth cues in a compact eyeglass form factor (Figure 7e). They used high-index glass-based monolithic metagrating couplers, and the chromatic aberration is compensated for by the whole system design rather than optimizing achromatic metagratings. The design of the full-color holographic AR glass was accomplished by co-designing the waveguide geometry with inverse-designed metasurface gratings and incorporating an artificial intelligence-driven propagation model to compensate for aberrations originating from physical imperfections and enhance three-dimensional digital holographic images for three different colors and planes with different depths [49].

On the other hand, S. Moon et al. proposed a very similar but somewhat different single-layer waveguide solution (Figure 7f). By utilizing inverse-designed achromatic metagratings designed to diffract RGB light at the same propagation angle, they enabled dispersion-free light transmission within a single 500 µm thick substrate, thereby significantly reducing the device thickness and weight [84]. Similarly, Z. Tian et al. presented a high-performance full-color AR display technology utilizing a high-refractive-index medium (*n* ≈ 1.9–2.0) (Figure 7g). They employed inverse-designed metasurface couplers that leverage different diffraction orders for each wavelength to effectively suppress chromatic aberration, securing a wide FOV exceeding 45° [85].

For clarification, here we offer a summarization of the advantages, disadvantages, and fabrication methods of the configuration.

Advantages:

(1) Extreme miniaturization: The “folding” of the optical path allows long focal lengths to be squeezed into a thin substrate, drastically reducing the physical track length (*z*-height).
(2) Monolithic alignment: Since all optical elements are fabricated on a single substrate using lithography, the lateral alignment between components is defined by the lithographic precision (nm scale), eliminating the need for complex and error-prone manual assembly of discrete lenses.
(3) Mechanical stability: The solid-state nature of the device makes it highly robust against vibrations and environmental factors compared to free-space optics.

Disadvantages:

(1) Efficiency accumulation: In systems with multiple bounces, the total efficiency is the product of the efficiency of each reflection and metasurface interaction. Therefore, even small losses per bounce can accumulate and lead to significant total insertion loss.
(2) Stray light and crosstalk: Light that is not perfectly controlled (e.g., zero-order diffraction or scattering) can propagate within the waveguide as stray light, potentially reducing image contrast or causing signal crosstalk.
(3) Limited FOV: The angular range is often constrained by the critical angle for TIR or the angular acceptance of the metasurfaces.

Fabrication Methods:

(1) Deposition: A high-refractive-index dielectric layer (e.g., a-Si, TiO_2_, and SiN) is deposited on a glass substrate.
(2) Patterning: The metasurface nanostructures are defined via lithography and etching (E-beam or DUV).
(3) Mirror/aperture formation: For folded cameras or spectrometers, metal layers (Au, Ag, and Al) are deposited and patterned to create mirrors and apertures, confining the light within the designed path.
(4) Passivation: In some designs, a cladding layer (e.g., SU-8 or SiO_2_) is added to protect the structures or provide a symmetric index environment.

### 3.3. Metalens Combined with Refractive Optics

The integration of metasurfaces with conventional refractive optics, often termed hybrid meta-optics, represents a paradigm shift that overcomes the intrinsic limitations of utilizing either technology in isolation. While refractive lenses offer high optical efficiency and large apertures, they are bulky and suffer from material-limited dispersion, difficulty in complex phase-encoding functions; conversely, single metalenses provide compact form factors and much degree of freedom in regard to phase-encoding capability but struggle with limited bandwidth and efficiency at larger scales. Hybrid systems exploit the synergistic relationship between the two: the refractive component provides the primary optical power and efficiency, while the metasurface acts as a versatile corrector that manipulates the wavefront to eliminate chromatic and monochromatic aberrations without adding significant weight or volume [42,44,88,89,90,91,92,93,94,95,96,97,98,99,100,101,102,103,104,105,106,107]. This “division of labor” allows for broadband achromatic performance and high NA imaging that would traditionally require complex, multi-element lens assemblies. Moreover, the hybridization strategy is also preferred for lens systems requiring large apertures, such as telephoto lenses with long focal lengths. Telescopes require high angular resolution, and image brightness is also a critical issue. Thus, large-aperture operation with control of chromatic aberration and moderate optical power can be achieved much easier in a hybrid system compared to the cascaded meta-optics systems discussed above.

**Figure 8:**
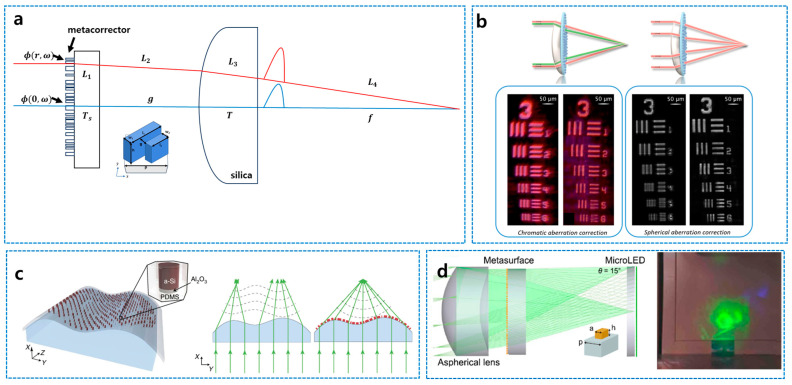
Hybrid refractive–meta-optics. (**a**) Metacorrector combined with a plano-convex lens to compensate spherical and chromatic aberrations [42]. (**b**) Large aperture-cemented doublet of metalens–spherical lens [88]. (**c**) Conformal metalens combined with curved refractive surface can extend design degree of freedom [44]. (**d**) Meta-aspheric doublet with a waveguide image combiner can project AR image from microLED display with the assistance of artificial neural network for higher-order aberration reduction [89]. Figure adapted with permission from (**b**) Ref. [88], Copyright 2021, R. Sawant et al.; (**c**) Ref. [44], Copyright 2015, S. M. Kamali et al., licensed under CC BY 4.0.; and (**d**) Ref. [89], Copyright 2024, American Chemical Society.

The first experimental work on this concept was proposed by W. T. Chen et al. [42]. As illustrated in Figure 8a, the metacorrector was introduced in combination with other refractive lenses for correcting chromatic and monochromatic aberrations. In their work, metacorrectors were cascaded with refractive lenses with a certain separation distance. On the other hand, in 2021, R. Sawant et al. proposed cemented doublet-based hybrid metalenses for large-scale (cm scale aperture) correction of longitudinal chromatic or spherical aberrations (Figure 8b) [88]. Furthermore, this hybrid approach enables the decoupling of optical function from geometrical form, as shown by S. M. Kamali et al. [44]. The work suggests the first experimental work of “conformal meta-optics” by developing conformal flexible metasurfaces that transform cylindrical substrates into aspherical lenses (described in Figure 8c), proving that optical performance need not be dictated by the physical shape required for aerodynamic or ergonomic integration. The key issue of the work is to transfer silicon meta-atoms into a flexible PDMS layer, and the work proved the near-unity yield of transfer (99.5%). It is expected that “conformal meta-optics” not only enables arbitrarily shaped metalenses but also enlarge the design degree of freedom of hybrid refractive–meta-optics by enabling demonstration of various cemented doublet or triplet hybrid lenses consisting of a metalens and curved refractive lenses.

Building on these physical advantages, recent developments have propelled hybrid meta-optics into the realm of computational and system-level optimization, enabling ultra-compact high-performance imaging devices. Advanced design frameworks now employ end-to-end differentiable optimization to jointly tune the physical parameters of the optics and the post-processing algorithms. For example, in the domain of AR, Q. Chen et al. (2024) demonstrated a compact AR display using a refractive–meta hybrid lens combined with neural network-based image reinforcement, achieving a wide FOV (30°) and low distortion (<2%, but monochromatic) while maintaining a minimal track length, illustrating the practical potential of hybrid meta-optics in next-generation wearable displays (Figure 8d) [89]. Q. Zhang et al. (2025) introduced a vectorial Generalized Snell’s Law-enabled differentiable ray tracing model to jointly optimize a large-aperture (8 mm) hybrid system, reducing chromatic aberration by 83% compared to aspheric singlets [90].

Besides the abovementioned representative papers, recently, there have been a variety of numerical and experimental studies to combine a thick-refractive-lens group with a dielectric metalens to compensate multiple aberrations in unprecedented or more compact and efficient ways [91,92,93,94,95,96,97,98,99,100,101,102,103,104,105,106,107].

In the field of hybrid refractive–meta-optics, one of the most critical challenges is the immense disparity in spatial scales between subwavelength meta-atoms and macroscopic refractive components. Establishing a unified framework capable of simultaneously designing and analyzing these multi-scale elements is essential for practical deployment. The recent literature proposes several methodologies to bridge this gap, each with distinct advantages and limitations regarding computational efficiency and physical accuracy.

For example, A. C. Cuillerier et al. (2023) proposed a method that integrates a semi-analytical model of dielectric nanostructures directly into commercial ray-tracing software (Zemax OpticStudio) via a dynamic link library (DLL) [100]. This approach allows for the rapid calculation of phase discontinuities induced by metasurfaces within a standard ray-tracing environment, enabling the use of built-in optimization algorithms for system-level design. However, since this method relies primarily on ray optics and the Generalized Snell’s Law, it fundamentally neglects diffraction effects and is limited to regimes where the metasurface-phase profile varies slowly.

Taking a more rigorous approach of physical optics, Shih and Renshaw (2025) developed a framework based on the Gaussian decomposition method combined with rigorous coupled-wave analysis (RCWA) [102]. By decomposing vector fields into Gaussian beamlets, this method can simulate complex wave propagation, including polarization effects and diffraction through refractive optics, which are often overlooked in scalar ray-field methods. Although this vector field framework provides a comprehensive full-wave solution essential for polarization-sensitive designs, it requires careful decomposition strategies and is computationally more intensive than ray-based alternatives.

The seamless efficient integration of advanced diffractive elements into standard optical design workflows is a prerequisite for the industrial adoption of hybrid optics. In this context, our group has recently demonstrated a significant milestone by developing an advanced, efficient sequential ray-tracing simulator capable of modeling multi-functional holographic optical elements (HOEs) within Zemax OpticStudio using a custom DLL [108]. This work successfully incorporated complex diffraction behaviors into a commercial design tool, enabling the practical optimization of multi-functional holographic systems by embedding an RCWA-based multiplexed HOE simulator in DLL. Drawing from this success, we suggest that future research in refractive–meta-holographic hybrid systems should similarly focus on developing robust, user-friendly simulation environments—such as enhanced DLLs or plug-ins—that can rigorously handle the unique phase and polarization characteristics of metasurfaces and whole lens systems while maintaining compatibility with the powerful optimization engines of existing optical design software based on geometrical and wave optics-based analysis.

### 3.4. In-Plane Spatial Multiplexing of Metalenses

Compared to the abovementioned compound architectures, in-plane spatial multiplexing involves arranging multiple metalens elements laterally on a single substrate; thus, this strategy aims to provide different imaging functionality based on sampling and Fourier transform. This architecture is particularly transformative for compact wide-field microscopy and computational imaging, where it enables the capture of diverse optical information—such as polarization states or light fields—simultaneously. There are several representative studies published so far that are noteworthy enough to be discussed in detail.

First, for 3D imaging, Zhang et al. (2025) demonstrated a 5-by-5 achromatic metalens array based on high-aspect-ratio Si_3_N_4_ nanostructures [109]. This system captures 25 distinct sub-images simultaneously to enable full-color light-field imaging with an average focusing efficiency of over 80% across the visible spectrum, successfully decoupling the trade-off between achromatic bandwidth and wide FOV in a chip-integrated form factor.

**Figure 9:**
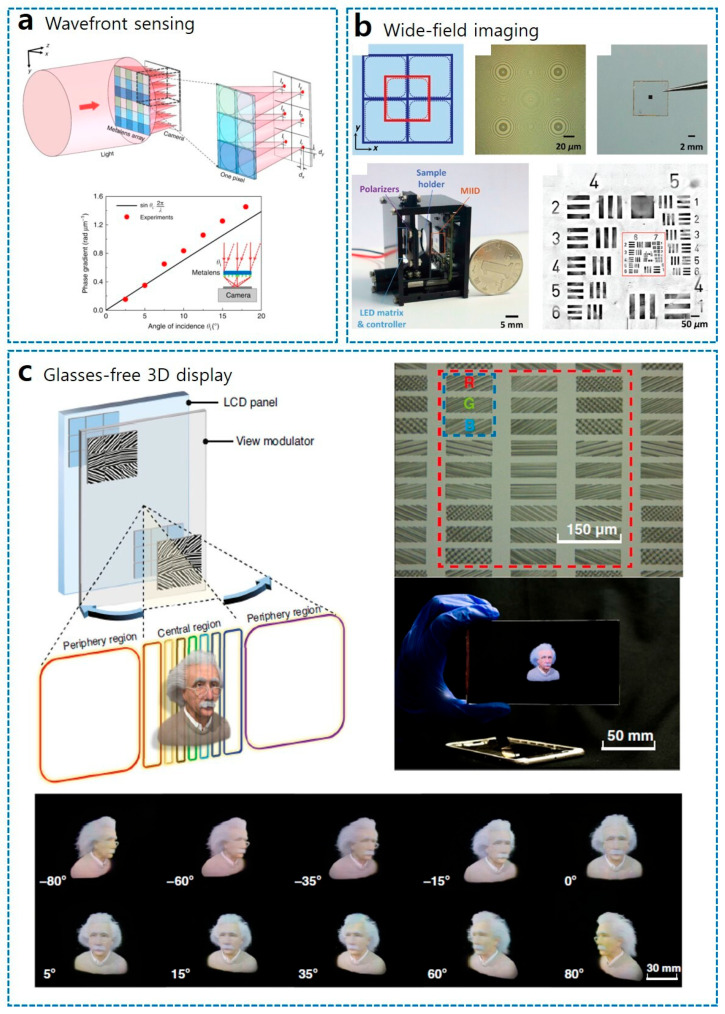
In-plane spatial multiplexing of metasurfaces for advanced imaging and display applications. (**a**) Polarization-dependent metalens array-based Shack–Hartmann wavefront sensor [110]. (**b**) Wide-FOV microscope with metalens array [111]. (**c**) Foveated glasses-free 3D display with spatially varying metagrating array [112]. Figure adapted with permission from (**a**) Ref. [110], Copyright 2018, Z. Yang et al.; (**b**) Ref. [111], Copyright 2020, B. Xu et al.; and (**c**) Ref. [112], Copyright 2021, J. Hua et al., licensed under CC BY 4.0.

A prominent implementation of in-plane spatial multiplexing for optical sensing is demonstrated by Z. Yang et al. (2018), who realized a generalized Hartmann–Shack detector capable of simultaneous full-Stokes polarimetry and wavefront sensing (Figure 9a) [110]. In this all-dielectric design, the unit cell is spatially multiplexed into a 2-by-3 sub-array of distinct silicon metalenses, where each sub-element is engineered to focus a specific polarization basis—encompassing linear, diagonal, and circular states—onto a common focal plane (Figure 9a). This configuration offers a significant advantage over traditional wavefront sensors by utilizing the foci intensities to reconstruct the complete local polarization state (Stokes parameters), while concurrently extracting phase gradients from the focal spot displacements. Consequently, this compact architecture enables real-time, comprehensive beam diagnostics for complex vector beams, such as radially or azimuthally polarized light, without requiring bulky moving parts or separate optical paths.

To address the “blind spots” typically found in conventional lens array stitching, B. Xu et al. (2020) developed a polarization-multiplexed dual-phase metalens array integrated directly into a CMOS sensor (Figure 9b) [111]. By exploiting the polarization sensitivity of the GP, the system switches between two complementary sets of lens arrays using left and right circular polarizations, effectively stitching a wide-field microscopic image without mechanical movement or resolution loss.

Beyond image capture, spatial multiplexing has also been ingeniously applied to light-field projection, specifically to resolve the critical trade-offs between spatial resolution and viewing angle in glasses-free 3D displays (Figure 9c). Conventional multi-view displays struggle to provide high angular resolution over a wide viewing zone due to limited total display bandwidth. Addressing this, J. Hua et al. (2021) proposed a “foveated” 3D display architecture enabled by a large-scale 2D-metagrating complex [112]. Inspired by the biological eye, this system spatially varies the information density across the display: it projects dense views with high angular resolution in the central zone for precise stereopsis, while distributing sparse views in the periphery to maximize the viewing angle. By utilizing efficient interference lithography to fabricate these large-area view modulators made of 2D-metagrating complex, the researchers achieved an LC display-based video-rate full-color 3D display with an unprecedented 160° horizontal viewing angle, demonstrating the scalability of multiplexed meta-optics for next-generation portable electronics. Moreover, there are numerous other works on in-plane spatially multiplexed metalens systems for applications such as phase imaging, polarization imaging, and so on [113,114,115,116,117,118,119,120,121,122,123,124].

### 3.5. Summary of Cascaded Metalens Systems

As discussed so far, the evolution of compound meta-optics has given rise to diverse architectural configurations for high-performance imaging applications, each tailored to address specific optical challenges while presenting unique engineering trade-offs. This section briefly summarizes the comparative advantages and limitations of the key categories discussed above:

(1) Metalens combined with an iris: By placing an aperture stop at the front focal plane, this configuration effectively eliminates off-axis aberrations such as coma and astigmatism, enabling FOV imaging. However, the introduction of the iris inherently restricts the entrance pupil diameter, thereby reducing the overall optical throughput and light-collection efficiency.
(2) Cascaded metalenses (separated substrates): Utilizing multiple independent metasurfaces increases the degrees of freedom required to correct chromatic aberrations and Petzval field curvature. While this approach offers high design flexibility, it incurs significant assembly challenges due to stringent lateral and axial alignment tolerances, alongside reduced transmission from multiple interfacial reflections.
(3) Double-sided metalens: Fabricating metalenses on both sides of a single transparent substrate offers a monolithic doublet solution that significantly relaxes alignment constraints and minimizes system volume. The primary disadvantage lies in the fabrication complexity, necessitating precise dual-side lithography and handling processes.
(4) Cladded bilayer metalens: This architecture achieves an ultra-compact, mechanically robust form factor by stacking layers directly with a spacer material. While it ensures precise inter-layer distance and stability, it involves a challenging multi-step fabrication process requiring rigorous planarization and material compatibility.
(5) Folded meta-optics: Leveraging polarization-dependent reflection to fold the optical path allows for long effective focal lengths within an extremely short physical track, ideal for compact telephoto systems. The trade-off is a notable reduction in radiometric efficiency due to multiple reflections and polarization filtering, along with susceptibility to ghosting artifacts.
(6) Hybrid refractive–meta-optics: This hybrid approach synergizes the high focusing power and efficiency of bulk refractive lenses with the superior aberration-correction capabilities of metalenses. Although it delivers high-performance achromatic imaging, it compromises the “flat optics” advantage by reintroducing bulk elements and complicating the integration of dissimilar optical components.
(7) In-plane spatially multiplexed metalens arrays: By interleaving or tiling distinct functional units on a single plane, this design enables parallel multichannel sensing (e.g., spectral or polarization sorting). However, this spatial division often results in a trade-off between the effective resolution per channel and inter-channel crosstalk.

## 4. Cascaded Metasurface System for Meta-Hologram and Optical Neural Network

### 4.1. Janus Meta-Hologram and Optical Encryption

In the broader landscape of compound meta-optics, cascaded metasurface systems for holography represent a distinct paradigm shift from their imaging counterparts. While general compound metalens systems primarily utilize multi-layer architectures to correct monochromatic and chromatic aberrations, expand the field of view, or enhance focusing efficiency, cascaded holographic systems prioritize the expansion of information capacity and optical security. By leveraging the propagation of light between distinct phase/amplitude masks, these systems introduce non-commutative optical transformations that are mathematically impossible in single-layer designs. This architecture allows for advanced functionalities, such as optical secret sharing, high-density multiplexing, and the breaking of optical symmetries to achieve Janus-like behaviors, where the system’s response varies drastically depending on the propagation direction or polarization state.

**Figure 10:**
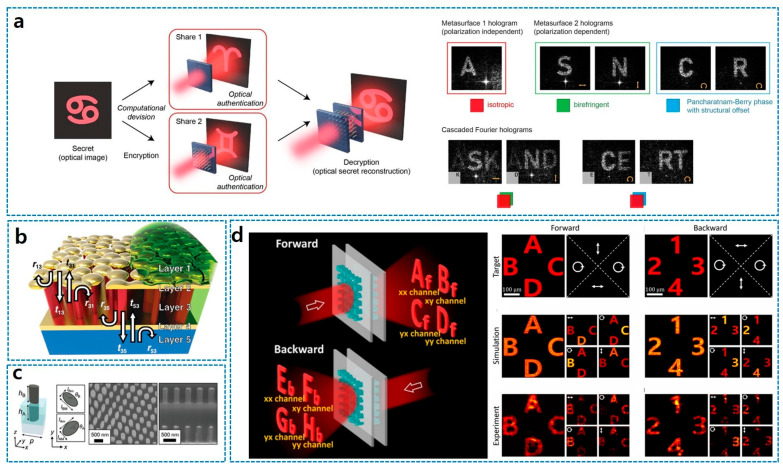
Janus meta-hologram applications consisting of cascaded metasurfaces. (**a**) Optical encryption and secret sharing [125] based on meta-hologram. (**b**) Tunable Janus effect of coloring in multi-layered metasurface [126]. (**c**) Extension of bidirectional asymmetry of meta-hologram by stacked Janus metasurfaces [127]. (**d**) Cascaded Janus metasurface for fully generalized bidirectional asymmetry of meta-hologram [128]. Figure adapted with permission from (**a**) Ref. [125], Copyright 2021, P. Georgi et al.; (**b**) Ref. [126], Copyright 2020, T. Kim et al.; and (**c**) Ref. [127], Copyright 2024, H. Kim et al., licensed under CC BY 4.0.; and (**d**) Ref. [128], Copyright 2025 American Chemical Society.

The evolution of this field is well-illustrated by the progression of the reviewed literature. P. Georgi et al. (2018) pioneered the concept of “optical secret sharing” using physically separable metasurfaces [125]. In their work, individual metasurfaces (shares) act as phase-only Fourier holograms that function as unique identifiers, but the encrypted “secret” image is only revealed when the shares are stacked with precise alignment, accumulating the phase shifts of both layers (Figure 10a). This work notably utilized the system’s high alignment sensitivity for spatial multiplexing. Shifting the focus to tunable reflective optics, as shown in Figure 10b, T. Kim et al. (2020) demonstrated an asymmetric optical camouflage system using a liquid-permeable Fabry–Perot etalon architecture, termed the “Bruggeman effective etalon” [126]. While structurally different from diffractive cascades, this metal–dielectric–metal stack achieves a tunable optical Janus effect where reflective colors and hidden messages change based on the viewing direction and the refractive index of infiltrated solvents.

Recent advancements have focused on overcoming the theoretical limits of reciprocity to achieve full bidirectional control. As described in Figure 10c, H. Kim et al. (2024) proposed a bilayer metasurface platform that generalizes asymmetric transmission for arbitrary input polarizations [127]. By mathematically elucidating the relationship between forward and backward Jones matrices and utilizing spatial partitioning of the transmission space, they successfully demonstrated polarization direction-multiplexed Janus vectorial holograms that generate four distinct independent images. Building upon this generalized framework, J. Bang et al. (2025) introduced a fully generalized cascaded meta-optics platform comprising physically separated dielectric metasurfaces to achieve fully generalized vectorial bidirectional asymmetry [128] (Figure 10d). Their work utilizes a stochastic gradient descent algorithm to optimize the inherent stop filtering effect-based non-commutative light propagation between layers, suggesting a generalized framework for complete and independent modulation of all eight parameters of the forward and backward Jones matrices. This allows for the realization of scalar and vectorial asymmetric holograms that relieve the inherent constraints of reciprocity found in single-layer systems.

### 4.2. Free-Space Meta-Optics Neural Network

The extension of cascaded diffractive optics into the realm of computational imaging has established the foundation for free-space optical neural networks (FSONNs), a paradigm pioneered by the seminal work by X. Lin et al. (2018) [129]. This research introduced the diffractive deep neural network, which utilized 3D-printed layers to modulate the phase of terahertz wave, effectively creating a physical embodiment of a deep-learning algorithm capable of performing inference at the speed of light.

**Figure 11:**
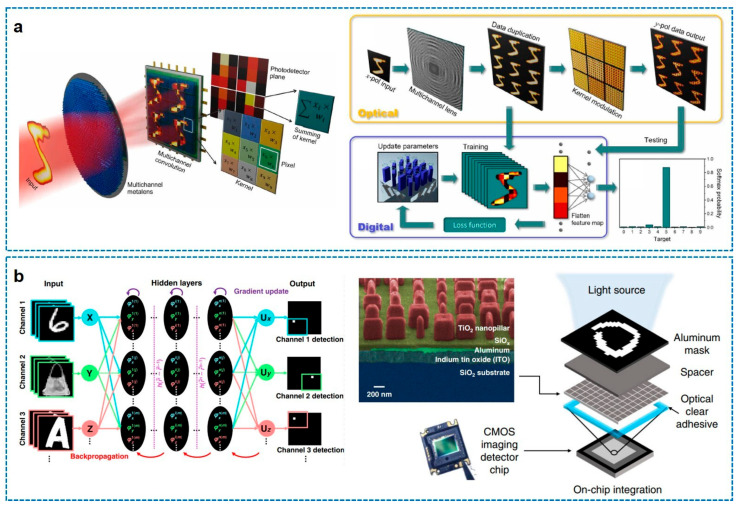
Free-space optical neural network applications using compound meta-optics. (**a**) Scheme and working principle of meta-optics object classifier. (**b**) Metasurface-based free-space optical neural networks demonstrated by on-chip integration with a CMOS image sensor array. Figure adapted with permission from (**a**) Ref. [48], Copyright 2022, H. Zheng et al.; and (**b**) Ref. [131] Copyright 2022, X. Luo et al., licensed under CC BY 4.0.

Although the initial implementation operated at longer wavelengths, it provided the essential architectural blueprint for subsequent advancements in the optical regime using metasurfaces. In this context, in the field of meta-optics, a different solution involving a free-space optical accelerator for object classification was proposed by H. Zheng et al. [48]. By taking advantage of the multi-functionality of metalenses and compound meta-optics, they demonstrated a significant leap by implementing physical convolution using a metasurface-based architecture (Figure 11a). They implemented a multi-functional doublet metalens-based meta-optics accelerator combined with an image sensor array. The first metalens was designed to make nine different foci via the spatial multiplexing strategy (supercell arrangement with nine meta-atoms). The second metalens was made to act as the specific kernel metasurface, generating unique feature maps through optical transmission, achieving high classification accuracy (~93%) on the MNIST dataset and validating the potential of metasurfaces to replace digital convolutional layers with passive optical elements.

More recently, the evolution of the previous system by H. Zheng et al. (2022) [48] has shifted toward multichannel meta-optics accelerators for incoherent light (H. Zheng et al., 2024) [130]. This study introduced angle and polarization-multiplexed doublet meta-imagers by engineering the point spread function to mimic convolutional operations and designing two opposite sign kernels depending on the handedness of circular polarization. By integrating these meta-optics accelerators with a polarization-sensitive photodetector array, the system successfully performed complex classification tasks, such as the Fashion-MNIST dataset, demonstrating theoretical and experimental accuracies comparable to traditional electronic networks (98.6 and 88.8% accuracy). These milestones—from the diffractive layers of X. Lin et al. [129] to the multichannel meta-imagers of H. Zheng et al. [48,130]—collectively illustrate the rapid maturation of compound meta-optics from simple wavefront shaping to sophisticated, energy-efficient computing engines.

While the pioneering frameworks established by X. Lin et al. and H. Zheng et al. successfully validated the computational versatility of diffractive and meta-optics layers, these architectures predominately relied on free-space propagation requiring substantial physical volume and precise optical alignment. Addressing the critical challenge of miniaturization, as described in Figure 11b, X. Luo et al. (2022) proposed a paradigm shift toward ultra-compact, chip-scale implementations with their development of a metasurface-enabled on-chip multiplexed diffractive neural network operating in the visible spectrum [131]. Unlike prior configurations that necessitated external lenses or extended diffraction distances, this architecture features a monolithic design where a polarization-multiplexed metasurface is directly bonded to a commercial CMOS image sensor using a micrometer-scale optical clear adhesive spacer. This seamless integration eliminates the need for bulky optical benches, effectively compressing the entire inference engine into a form factor compatible with standard semiconductor electronics. Furthermore, this study distinguished itself by leveraging the high neuron density of subwavelength structures to enable sophisticated multitasking directly at the sensor level. By exploiting the birefringence of the TiO_2_ nanopillars, the system realizes a polarization-multiplexing scheme capable of executing distinct classification tasks—such as recognizing handwritten digits and fashion items concurrently—within a single compact device. This capability represents a significant advancement over the single-task, volumetric setups of earlier milestones, as it demonstrates that massive optical parallelism can be harnessed in a portable, energy-efficient architecture. Consequently, this work bridged the gap between theoretical meta-optics and practical deployment, offering a scalable pathway for integrating intelligent machine vision capabilities directly into the next generation of edge-computing hardware.

It should be noted that this section highlights specific FSONN implementations primarily to illustrate the evolution of compound meta-optics designs, rather than providing a rigorous survey of the field of optical neural networks. For a more detailed analysis of the fundamental principles and broader architectural diversity of free-space optical neural networks, readers are directed to recent comprehensive reviews dedicated to this subject [132,133,134,135].

## 5. Conclusions and Outlook

In conclusion, the paradigm of compound meta-optics represents a pivotal evolution in optical engineering, transitioning from isolated diffractive elements to sophisticated, multi-component architectures that rival or supplement the performance of bulky refractive systems. As explored in this review, this transition is fundamentally reshaping the landscape of imaging, display, sensing, and computing via optical technologies. By rigorously correcting monochromatic or chromatic aberrations and expanding various performance metrics such as FOV, compound meta-optics is enabling the miniaturization of high-performance lens systems, including cameras, microscopes, telescopes, and sensors, without compromising image quality.

Furthermore, the capability to manipulate light with high degrees of freedom unlocks innovative applications, including ultra-compact AR/VR displays, machine vision tasks, advanced bio-sensing interfaces, optical information security, and analog optical computing for artificial intelligence. Although challenges in large-scale fabrication and precise system integration remain to be fully addressed, the convergence of deep learning-based inverse design and active materials promises to accelerate the practical adoption of these technologies. Ultimately, compound meta-optics is poised to become a cornerstone of next-generation optical devices, driving transformative advancements in consumer electronics, medical diagnostics, and autonomous or smart systems depending on hybrid optical–electronic hardware.

## Data Availability

Data are contained within the article.
